# Temporal trends in single and multiple organ fibrosis prevalence and primary care consultations: a population-based cohort study of 5.8 million individuals in England

**DOI:** 10.1136/bmjopen-2025-113800

**Published:** 2026-09-22

**Authors:** Georgie May Massen, Gisli Jenkins, Richard J Allen, Iain Stewart, Chris J Scotton, Rachel Chambers, Hilary Longhurst, Louise V Wain, Vidya Navaratnam, Jennifer K Quint, on behalf of DEMISTIFI Consortium

**Affiliations:** 1 School of Public Health, Imperial College London, London, UK; 2 National Heart and Lung Institute, Imperial College London, London, UK; 3 Division of Public Health and Epidemiology, University of Leicester, Leicester, England, UK; 4 NIHR Leicester Biomedical Research Centre, Leicester, England, UK; 5 National Heart and Lung Institute, Imperial College London, London, England, UK; 6 Faculty of Health Sciences, University of Exeter, Exeter, England, UK; 7 Department of Respiratory Medicine (UCL Respiratory), University College London, London, England, UK; 8 Dyskeratosis Congenita (DC) Action, London, UK; 9 Division of Public Health and Epidemiology, University of Leicester, Leicester, Leicestershire, UK; 10 The Institute for Lung Health, Leicester NIHR Biomedical Research Centre, University of Leicester, Leicester, Leicestershire, UK; 11 Institute for Respiratory Health, The University of Western Australia, Perth, Western Australia, Australia; 12 Faculty of Medicine, Imperial College London, London, UK

**Keywords:** Fibrosis, EPIDEMIOLOGY, Electronic Health Records, Prevalence

## Abstract

**Abstract:**

**Objective:**

To understand how prevalence estimates of single and multiple organ fibrosis have changed from 2012 to 2022.

**Design:**

Retrospective population-based cohort study.

**Setting:**

Data from primary (Clinical Practice Research Datalink Aurum) and secondary care (Hospital Episode Statistics Admitted Patient Care) electronic health records were used to conduct this study.

**Participants:**

Adults aged 18 years and older whose primary and secondary care records were available for research.

**Main outcome measure:**

Fibrotic conditions previously determined from a Delphi survey of clinicians; diagnoses were found in either primary or secondary care records. The primary analysis estimated the prevalence of both single and multiple organ fibrosis. A secondary analysis used Cox proportional hazards models to investigate the association between the time-updated number of fibrotic conditions and risk of death, adjusting for age and sex.

**Results:**

The cohort consisted of 5 839 459 people with at least one fibrotic condition and a denominator of 18 784 962 people. Over the study period, prevalence of fibrotic conditions increased by 6.72%, and as of 2022, 19.95% (95% CI 19.92% to 19.98%) of adults had at least one fibrotic condition. The prevalence of multiple organ fibrosis increased from 4.78% (95% CI 4.76% to 4.79%) in 2012 to 8.51% (95% CI 8.50% to 8.53%) in 2022. In the year preceding diagnosis of single organ fibrosis, the median number of primary care consultations was 14 compared with 21 consultations for people with multiple organ fibrosis. Compared with people with single organ fibrosis, people with two fibrotic conditions had a mortality HR of 2.90 (95% CI 2.87 to 2.93), while people with three fibrotic conditions had a mortality HR of 5.22 (95% CI 5.14 to 5.30).

**Conclusions:**

The prevalence of single and multiple organ fibrosis has substantially increased over a decade. People with multiple organ fibrosis access healthcare more than people with single organ fibrosis and are at a greater risk of mortality. Multiple organ fibrosis is estimated to affect more than 3 million people in the UK and is increasing. Better understanding of patterns of fibrotic multimorbidity may help to aid earlier diagnosis and opportunities for reducing mortality.

**STRENGTHS AND LIMITATIONS**

- This study used a large, representative primary care database linked to secondary care records, enabling identification of fibrotic conditions recorded across both healthcare settings.
- Fibrotic conditions were identified using previously published clinical code definitions, although some included conditions may only involve fibrosis at particular stages of disease.
- Regional prevalence estimates were standardised using the age and sex distribution of the 2021 England Census population to improve population representativeness.
- Ethnicity, deprivation and lifestyle factors such as smoking, body mass index and alcohol consumption could not be fully incorporated because sufficiently complete or comparable data were unavailable.
- Reliance on routinely recorded clinical coding may have resulted in misclassification or under-ascertainment of fibrotic conditions.

## Introduction

 Multiple long-term conditions (MLTCs; previously referred to as multimorbidity) have been defined as a research priority as they are associated with decreased quality of life and increased risk of mortality, more specifically premature death.^1–5^ Physical multimorbidity is associated with increased risk of new disease onset and worsening of existing mental health disorders.^6^ Additionally, MLTCs are more common in older people and are associated with decreased quality of life.^1 7^ It is predicted that by 2065, 26% of the population of England will be aged 65 years or older.^8 9^ Given this changing distribution of age, it is important that healthcare systems adapt to best serve the population and are prepared for the potential impacts of the changing population structure.

Fibrotic conditions can occur in any organ; they are characterised by excessive uncontrolled deposition of extracellular matrix which alters the tissues’ extracellular environment and can lead to organ failure.^10^ Historically, it has been thought that fibrosis contributes to around 45% of deaths in the industrialised world, with more recent estimates demonstrating that in a random sample of UK adults who died, 34.82% had at least one fibrotic condition.^11 12^

It is hypothesised that fibrosis is a biological mechanism which can affect multiple organs or lead to a cascade of fibrotic conditions on the development of an initial fibrotic condition. However, currently most research focuses on specific individual fibrotic conditions rather than the pathogenesis of fibrosis across organs. Therefore, little data exists on multiple organ fibrosis or the prevalence of multiple fibrotic conditions. A single study exists which estimated the prevalence of multiple organ fibrosis as of 2015 in England; 21.46% of adults in England had at least one fibrotic condition, while 6% had multiple organ fibrosis.^12^

Given the previously observed high prevalence of fibrotic conditions and the association between the number of fibrotic conditions and mortality, it is imperative to better understand the burden of fibrotic disease. This will provide evidence to support further research investment as well as healthcare service provision.

## Methods

We used the Clinical Practice Research Datalink (CPRD) Aurum, a nationally representative database of anonymised primary care electronic healthcare records. CPRD Aurum contains the data of around 24% of the UK population and is representative of the UK population with regard to age, sex and deprivation.^13 14^ Linked secondary care data from Hospital Episode Statistics (HES) were provided for this study by CPRD.

### Study population

The December 2024 build of CPRD Aurum was used in this analysis and was linked with HES Admitted Patient Care (APC) data. To calculate the prevalence of individual fibrotic conditions, single organ fibrosis and fibrotic multimorbidity, the denominator was derived from the whole CPRD population using the denominator files. To be eligible for inclusion in the denominator, people had to be either male or female (to ensure data completeness), over the age of 18 and enrolled at a practice which was eligible for linkage to HES APC, the denominator was generated per year and further stratified by age and sex where necessary. Broadly, the numerator included people who had a diagnosis of a fibrotic condition present in either their primary or secondary care, and whose records were eligible for linkage to HES APC. This was a rolling cohort, therefore entry was defined as the latest of registration start date plus 1 year, the start of the study (1 January 2012) and date of 18th birthday, while the date of study exit was defined as the earliest of the registration end date, the last collection date of data, date of death, end of HES linkage and the end of the study (31 December 2022).

### Outcomes of interest

Fibrotic conditions were previously defined in a Delphi study consisting of three survey rounds using clinical consensus as an indicator of whether a condition was fibrotic.^15^ Additionally, evidence has shown that treatment-resistant hypertension and insulin resistant type 2 diabetes (respectively) are associated with fibrotic conditions (compared with treated hypertension and non-complicated type 2 diabetes),^16 17^ indicating these more specific definitions of these common conditions (which were classified as fibrotic by clinicians within the Delphi survey) may be appropriate in defining forms of these conditions where fibrosis is present. These definitions were therefore used in addition to the other results of the Delphi survey. The following organ/disease groups were employed: atherosclerosis, bile duct fibrosis, blood vessel fibrosis, cardiomyopathy, fibrosis of the cardiac valves, insulin-resistant type 2 diabetes, integumentary fibrosis, intestinal/pancreatic fibrosis, liver fibrosis, lung fibrosis, lymphatic fibrosis, treatment resistant hypertension, reproductive fibrosis, skeletal fibrosis, systemic fibrosis and urinary tract fibrosis. Conditions were defined using both primary and secondary care records and the associated codelists can be viewed at https://github.com/NHLI-Respiratory-Epi/Epidemiology-of-single-and-multiple-organ-fibrosis. Each codelist comprised standardised clinical codes (medcodeid/ICD10) and their corresponding clinical descriptors. Outcomes were identified through exact matching of the coded medcodeid/ICD10 value recorded in the CPRD Aurum observation dataset or the HES APC dataset.

Secondary outcomes of interest were the number of fibrotic conditions (specifically the number of affected organ/disease groups, thereby removing the potential for double counting of overlapping conditions or conditions affecting the same organ) (1 organ, 2, 3, >=4, grouped as four or more due to low numbers) and mortality (defined using provided date of death by the Office for National Statistics (ONS)).

### Variables of interest

Other included variables of interest were the co-occurrence of fibrotic conditions, specifically which affected organ/disease groups were observed in the same individual (if fibrotic multimorbidity was present). Healthcare utilisation trends were also explored, with the number of primary care consultations in the years prior to and post diagnosis of fibrotic conditions. Routine visits, such as vaccinations and health checks, did not contribute towards these counts.

### Statistical analysis

We calculated the point prevalence of fibrosis (affecting at least one organ), single organ fibrosis and multiple organ fibrosis, as of 1 July, between the years of 2012 and 2022. Date of single organ fibrosis was defined as the date of diagnosis of first fibrotic condition in either CPRD Aurum or HES APC. When calculating the prevalence of single organ fibrosis, cases were censored at the date of multiple organ fibrosis. Date multiple organ fibrosis was defined as the date of diagnosis (first record) of a second fibrotic condition (affecting a different body system) in either primary or secondary care records. As a result of this censoring, the denominators used to estimate the prevalence of fibrosis, single organ fibrosis and multiple organ fibrosis differed; consequently, these prevalence estimates are not expected to sum to the overall prevalence of fibrosis affecting at least one organ. Furthermore, we calculated the prevalence of multiple organ fibrosis stratified by the number of fibrotic organs (two, three, four and five plus), these estimates were further stratified by sex. Prevalence estimates of fibrosis, single organ fibrosis and multiple organ fibrosis were calculated by region (North East, North West, Yorkshire and the Humber, East Midlands, West Midlands, East of England, London, South East, South West). To account for differences in population structure between regions, regional prevalence estimates were directly standardised by age and sex to the 2021 England Census population.^18^ Age-specific and sex-specific prevalence estimates were calculated within each region and weighted according to the corresponding age-specific and sex-specific distribution of the standard population. Additionally, prevalence estimates of single and multiple organ fibrosis were stratified by sex (female/male) and age (18–24, 25–29, 30–34, 35–39, 40–44, 45–49, 50–54, 55–59, 60–64, 65–69, 70–74, 75–79, 80–84, 85–89, 90+). We further calculated and report the fold change in prevalence between 2012 and 2022.

We looked to understand how healthcare utilisation differed in people with single organ fibrosis compared with multiple organ fibrosis to capture the burden of fibrotic conditions on healthcare. Using both primary care consultations and hospital admissions, we report the median number of each, relative to the year of single or multiple organ fibrosis diagnosis. Number of consultations/hospitalisations are reported (where data are available) for the 10 years prior to and post diagnosis.

Using the data of people with multiple organ fibrosis, we report the percentage of people with the 10 most commonly co-occurring fibrotic conditions. These co-occurring diseases were reported for everyone with multiple organ fibrosis and were also stratified by sex.

Furthermore, we calculated the point prevalence of fibrotic conditions (lung fibrosis, urinary tract fibrosis, liver fibrosis, insulin-resistant type 2 diabetes, blood vessel fibrosis, atherosclerosis, bile duct fibrosis, cardiomyopathy, integumentary fibrosis, intestinal/pancreatic fibrosis, lymphatic fibrosis, reproductive fibrosis, skeletal fibrosis, systemic fibrosis, treatment-resistant hypertension and fibrosis of the cardiac valves) as of 1 July 2012 and 2022, respectively. These estimates were additionally stratified by sex. Finally, using data from the 2021 Census, we used the number of people aged 17+ as of 2021 and multiplied this by the estimated prevalences of fibrosis, single organ fibrosis, multiple organ fibrosis and individual fibrotic conditions to estimate the number of people with these conditions as of 2022.^19^

Cox proportional hazards models were used to estimate the association between the number of fibrotic conditions and risk of mortality. A time updated exposure of the number of fibrotic conditions was used and models were adjusted for age and sex; the reference category was people with single organ fibrosis.

All prevalence estimates are reported alongside 95% CIs for clarity. All analyses were completed using Stata MP V.18.^20^ Full details are available at the end of the paper.

## Results

**Figure 1:**
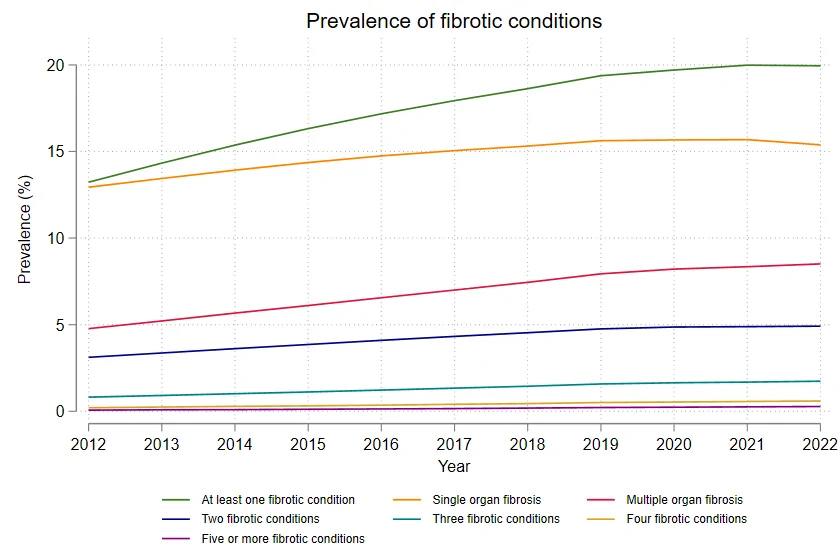
Prevalence of fibrotic conditions between 2012 and 2022. Prevalence estimates were calculated using outcome-specific denominators that varied according to the censoring applied. Individuals who developed multiple organ fibrosis were no longer considered at risk of single organ fibrosis and were therefore removed from the denominator for subsequent single organ fibrosis estimates. Consequently, prevalence estimates for the categories shown are not expected to sum to the overall prevalence of at least one fibrotic condition.

A cohort of 5 839 459 people with fibrosis was derived and included in this study. The denominator contained the information of 18 784 962 people (online supplemental table S1). The estimated prevalence of people with fibrotic conditions (irrespective of number) increased from 13.23% (95% CI 13.21% to 13.255) in 2012 to 19.95% (95% CI 19.92% to 19.98%) in 2022 (figure 1, online supplemental table S1). The estimated prevalence of single organ fibrosis increased from 12.94% (95% CI 12.92% to 12.97%) in 2012 to 15.39% (95% CI 15.39% to 15.41%) in 2022. In comparison, as of 2022, the estimated prevalence of multiple organ fibrosis was 8.51% (95% CI 8.50% to 8.53%). When further calculating the prevalence of multiple organ fibrosis, separating by the number of fibrotic conditions, reveals that the estimated prevalence (irrespective of number of conditions) has risen over the observed period (online supplemental table S2). Age and sex-standardised prevalence varied across regions of England (table 1). In 2022, the prevalence of at least one fibrotic condition was highest in the North East (26.54%, 95% CI 26.40% to 26.69%) and lowest in London (29.13%, 95% CI 20.0% to 20.18%) (online supplemental table S3). A similar pattern was observed for single organ fibrosis, the prevalence ranged from 18.04% (95% CI 17.99% to 18.10%) in London to 23.10% (95% CI 22.95% to 23.25%) in the North East. Multiple organ fibrosis showed greater regional variation, the highest prevalence was observed in the North East (10.80% (95% CI 10.69% to 10.90%) compared with 7.51% (95% CI 7.38% to 7.64%) in the East Midlands, where prevalence was lowest). Age and sex-standardised prevalence increased between 2012 and 2022 across all regions for each of the three fibrosis outcomes.

**Table 1:** Age and sex-standardised prevalence of fibrotic conditions, single organ fibrosis and multiple organ fibrosis, stratified by region

	At least one fibrotic condition, % (95% CI)			Single organ fibrosis, % (95% CI)			Multiple organ fibrosis, % (95% CI)		
	**2012**	**2017**	**2022**	**2012**	**2017**	**2022**	**2012**	**2017**	**2022**
North East	20.42 (20.28 to 20.55)	24.54 (24.40 to 24.67)	26.54 (26.40 to 26.69)	14.96 (14.83 to 15.09)	20.27 (20.13 to 20.41)	23.10 (22.95 to 23.25)	6.39 (6.30 to 6.47)	8.71 (8.71 to 8.80)	10.80 (10.69 to 10.90)
North West	18.10 (18.05 to 18.16)	22.25 (22.20 to 22.31)	23.55 (23.50 to 23.61)	13.47 (13.42 to 13.53)	18.45 (18.40 to 18.51)	20.60 (20.54 to 20.66)	5.49 (5.46 to 5.53)	8.02 (7.99 to 8.06)	9.62 (9.58 to 9.66)
Yorkshire	17.50 (17.38 to 17.63)	21.18 (21.06 to 21.31)	22.52 (22.37 to 22.68)	13.49 (13.37 to 13.61)	17.92 (17.80 to 18.05)	20.00 (19.84 to 20.15)	4.84 (4.77 to 4.91)	6.93 (6.85 to 7.00)	8.50 (8.39 to 8.60)
East Midlands	16.03 (15.88 to 16.19)	19.71 (19.56 to 19.86)	19.70 (19.50 to 19.89)	12.35 (12.20 to 12.50)	16.68 (16.53 to 16.84)	17.62 (17.42 to 17.81)	4.52 (4.43 to 4.61)	6.52 (6.42 to 6.61)	7.51 (7.38 to 7.64)
West Midlands	16.21 (16.15 to 16.27)	20.25 (20.19 to 20.31)	21.97 (21.91 to 22.02)	12.79 (12.73 to 12.84)	17.34 (17.28 to 17.40)	19.64 (19.58 to 19.70)	4.41 (4.38 to 4.44)	6.73 (6.69 to 6.77)	8.30 (8.26 to 8.34)
East of England	16.17 (16.07 to 16.28)	20.14 (20.03 to 20.25)	21.58 (21.47 to 21.70)	12.63 (12.52 to 12.73)	17.26 (17.15 to 17.37)	19.44 (19.32 to 19.56)	4.31 (4.25 to 4.37)	6.47 (6.40 to 6.54)	7.98 (7.91 to 8.07)
London	16.19 (16.13 to 16.25)	19.50 (19.44 to 19.56)	20.13 (20.07 to 20.18)	12.47 (12.41 to 12.52)	16.57 (16.51 to 16.63)	18.04 (17.99 to 18.10)	4.98 (4.94 to 5.02)	7.08 (7.04 to 7.12)	8.25 (8.21 to 8.30)
South East	15.68 (15.63 to 15.73)	19.24 (19.18 to 19.29)	20.61 (20.56 to 20.66)	12.23 (12.18 to 12.27)	16.44 (16.39 to 16.49)	18.47 (19.42 to 18.52)	4.31 (4.25 to 4.37)	6.23 (6.20 to 6.26)	7.71 (7.67 to 7.74)
South West	16.66 (16.60 to 16.73)	20.16 (20.10 to 20.23)	21.73 (21.66 to 21.81)	12.76 (12.69 to 12.82)	17.06 (16.99 to 17.13)	19.32 (19.25 to 19.39)	4.73 (4.69 to 4.77)	6.67 (6.63 to 6.71)	8.25 (8.20 to 8.30)

Prevalence estimates are presented as percentages with 95% CIs and were directly standardised by age and sex to the 2021 England Census population.

**Figure 2:**
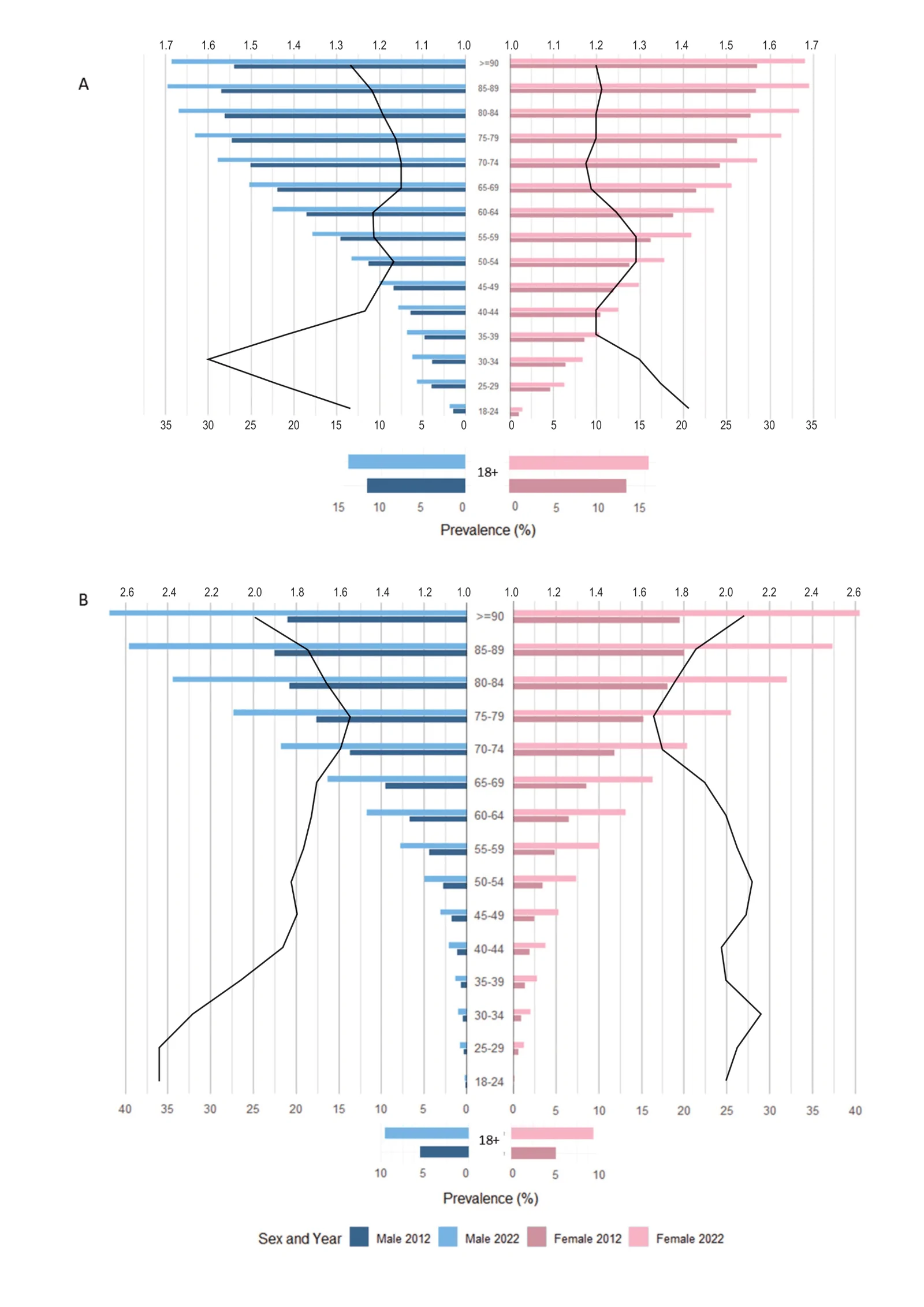
Comparative prevalence of (A) single organ fibrosis and (B) multiple organ fibrosis in people stratified by age and sex as of 2012 and 2022**.** The black line indicates the fold change in prevalence.

The prevalence of single and multiple organ fibrosis was observed to be greater in females compared with males (multiple organ fibrosis prevalence 2022: female 9.37%, 95% CI 9.34 to 9.39, male 7.66%, 95% CI 7.64 to 7.68) (figure 2, online supplemental tables S4, S5). The prevalence of single and multiple organ fibrosis increased with respect to increasing age. It was observed that males aged 30–34 years had the greatest fold change (1.59) in single organ fibrosis prevalence between 2012 and 2022. Overall, a greater fold change in multiple organ fibrosis (range: 1.56–2.44) was observed compared with single organ fibrosis (range: 1.15–1.59).

**Figure 3:**
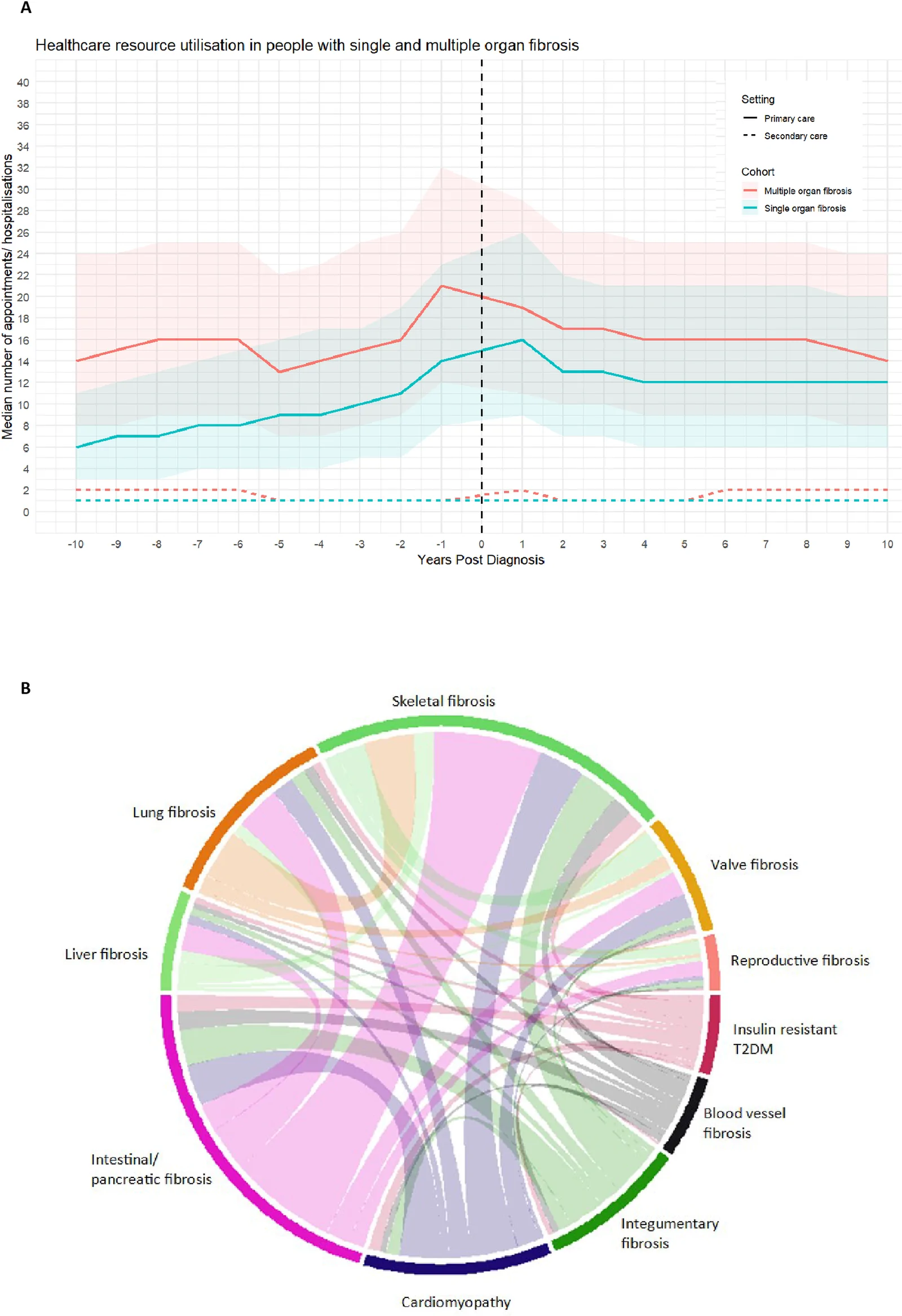
(A) Healthcare utilisation in people with single and multiple organ fibrosis with respect to the year of diagnosis. The shaded region reflects the IQRs of the number of primary care consultations. (B) Circular plot of co-occurring fibrotic diseases. Width of connections is proportional to the number of people with disease combinations. T2DM, type 2 diabetes mellitus.

Healthcare resource utilisation increased in the years leading up to diagnosis of either single organ or multiple organ fibrosis (figure 3A). In the year preceding diagnosis of single organ fibrosis, the median number of primary care consultations was 14 (IQR: 8–23), while the median number over the same period in people with multiple organ fibrosis was 21 (IQR: 12–32) consultations. Healthcare utilisation subsequently declined following diagnosis in both groups.

Investigating the data of people with multiple fibrotic conditions revealed commonly co-occurring disease pairs (figure 3B). The 10 most commonly co-occurring diseases can be found in table 2, the most common combination was intestinal/pancreatic fibrosis alongside skeletal fibrosis which was found in over one-quarter (28.96%) of the records of people with multiple organ fibrosis, a larger percentage of females (33.04%) had this combination compared with males (24.17%). The second most commonly co-occurring diseases in males was cardiomyopathy and skeletal fibrosis which was identified in 16.16% of the records of males with multiple organ fibrosis; in comparison, this combination was found to affect 9.38% of females (and was the sixth most common). The second most common combination of conditions in females was lung fibrosis alongside skeletal fibrosis (13.53%), a similar percentage of males had this disease combination (13.08%).

**Table 2:** The 10 most common disease pairs identified in the data of people with multiple organ fibrosis, stratified by sex

Rank	Overall (N, %)	Male (N, %)	Female (N, %)
**1**	Intestinal/pancreatic fibrosis and skeletal fibrosis (*586 287, 28.96*)	Intestinal/pancreatic fibrosis and skeletal fibrosis (*225 011, 24.17*)	Intestinal/pancreatic fibrosis and skeletal fibrosis (*361 276, 33.04*)
**2**	Lung fibrosis and skeletal fibrosis (*269 663, 13.32*)	Cardiomyopathy and skeletal fibrosis (*150 401, 16.16*)	Lung fibrosis and skeletal fibrosis (*147 927, 13.53*)
**3**	Cardiomyopathy and skeletal fibrosis (*252 957, 12.50*)	Cardiomyopathy and intestinal/pancreatic fibrosis (*137 424, 14.76*)	Integumentary fibrosis and skeletal fibrosis (*142 302, 13.01*)
**4**	Intestinal/pancreatic fibrosis and lung fibrosis (*244 320, 12.07*)	Lung fibrosis and skeletal fibrosis (*121 736, 13.08*)	Intestinal/pancreatic fibrosis and lung fibrosis (*125 717, 11.50*)
**5**	Intestinal/pancreatic fibrosis and cardiomyopathy (*225 185, 11.12*)	Intestinal/pancreatic fibrosis and lung fibrosis (*118 603, 12.74*)	Integumentary fibrosis and intestinal/pancreatic fibrosis (*122 666, 11.22*)
**6**	Integumentary fibrosis and skeletal fibrosis (*218 330, 10.78*)	Cardiomyopathy and lung fibrosis (*80 127, 8.61*)	Cardiomyopathy and skeletal fibrosis (*102 556, 9.38*)
**7**	Integumentary and intestinal fibrosis/pancreatic fibrosis (*193 196, 9.54*)	Cardiomyopathy and valve fibrosis (*79 929, 8.59%*)	Biliary fibrosis and intestinal/pancreatic fibrosis (*88 595, 8.10*)
**8**	Skeletal fibrosis and valve fibrosis (*154 086, 7.61*)	Integumentary fibrosis and skeletal fibrosis (*76 028, 8.17*)	Cardiomyopathy and intestinal/pancreatic fibrosis (*87 761, 8.03*)
**9**	Intestinal/pancreatic fibrosis and liver fibrosis (*149 564, 7.39*)	Intestinal/pancreatic fibrosis and liver fibrosis (*75 544, 8.12*)	Skeletal fibrosis and valve fibrosis (*86 840, 7.94*)
**10**	Biliary fibrosis and intestinal/pancreatic fibrosis (*138 378, 6.84*)	Integumentary fibrosis and intestinal/pancreatic fibrosis (*70 530, 7.58*)	Intestinal/pancreatic and liver fibrosis (*74 020, 6.77*)

The prevalence of all fibrotic conditions (except urinary tract fibrosis) increased over the observed decade. As of 2022, the five most prevalent fibrotic conditions were intestinal/pancreatic fibrosis, skeletal fibrosis, integumentary fibrosis, cardiomyopathy and reproductive fibrosis (table 3). The conditions which were more prevalent in females compared with males were skeletal fibrosis, intestinal/pancreatic fibrosis, integumentary fibrosis, reproductive fibrosis, biliary fibrosis, blood vessel fibrosis, systemic fibrosis and treatment resistant hypertension. However, the following conditions were more common in males: cardiomyopathy, lung fibrosis, liver fibrosis, valve fibrosis, insulin-resistant type two diabetes, urinary fibrosis, atherosclerosis and lymphatic fibrosis.

**Table 3:** Prevalence of fibrotic conditions as of 2012 and 2022, stratified by sex

Condition	Whole cohort 2012	Whole cohort 2022	Female 2012	Female 2022	Male 2012	Male 2022
Atherosclerosis	0.38 (0.37–0.38)	0.56 (0.56–0.57)	0.29 (0.28–0.29)	0.44 (0.43–0.44)	0.47 (0.46–0.47)	0.69 (0.68–0.70)
Biliary fibrosis	0.66 (0.65–0.66)	1.00 (0.99–1.00)	0.91 (0.90–0.92)	1.39 (1.38–1.40)	0.39 (0.39–0.40)	0.60 (0.60–0.61)
Blood vessel fibrosis	1.18 (1.17–1.18)	1.22 (1.21–1.23)	1.32 (1.31–1.33)	1.31 (1.30–1.32)	1.02 (1.01–1.03)	1.13 (1.12–1.14)
Cardiomyopathy	2.44 (2.43–2.45)	3.00 (2.99–3.01)	1.43 (1.42–1.44)	1.86 (1.85–1.87)	3.47 (3.45–3.49)	4.14 (4.12–4.15)
Insulin resistant T2DM	1.05 (1.04–1.05)	1.17 (1.17–1.18)	0.97 (0.96–0.98)	1.15 (1.14–1.16)	1.12 (1.11–1.13)	1.20 (1.19–1.21)
Inflammatory fibrosis	2.95 (2.94–2.96)	3.84 (3.83–3.85)	3.22 (3.21–3.24)	4.39 (4.37–4.41)	2.66 (2.65–2.68)	3.28 (3.27–3.30)
Intestinal/pancreatic fibrosis	4.23 (4.21–4.24)	8.15 (8.14–8.17)	4.96 (4.94–4.98)	9.34 (9.31–9.36)	3.47 (3.45–3.49)	6.97 (6.95–6.99)
Liver fibrosis	0.62 (0.62–0.63)	1.63 (1.62–1.64)	0.51 (0.51–0.52)	1.59 (1.58–1.60)	0.74 (0.73–0.74)	1.67 (1.66–1.68)
Lung fibrosis	0.96 (0.95–0.96)	1.63 (1.63–1.64)	0.87 (0.87–0.88)	1.55 (1.54–1.56)	1.04 (1.03–1.05)	1.71 (1.70–1.72)
Lymphatic fibrosis	0.20 (0.20–0.21)	0.30 (0.30–0.30)	0.19 (0.18–0.19)	0.29 (0.28–0.29)	0.22 (0.21–0.22)	0.32 (0.31–0.32)
Reproductive fibrosis	1.64 (1.63–1.65)	2.16 (2.15–2.17)	2.37 (2.35–2.38)	3.12 (3.10–3.13)	0.90 (0.89–0.91)	1.21 (1.20–1.22)
Skeletal fibrosis	5.26 (5.24–5.27)	8.01 (7.99–8.03)	6.25 (6.22–6.27)	9.42 (9.39–9.45)	4.24 (4.22–4.26)	5.60 (5.58–5.62)
Systemic fibrosis	0.40 (0.40–0.41)	0.58 (0.57–0.58)	0.50 (0.50–0.51)	0.72 (0.71–0.72)	0.30 (0.30–0.31)	0.43 (0.43–0.44)
Resistant hypertension	0.13 (0.13–0.13)	0.24 (0.24–0.24)	0.14 (0.14–0.15)	0.28 (0.27–0.28)	0.12 (0.11–0.12)	0.20 (0.20–0.21)
Urinary fibrosis	0.82 (0.81–0.82)	0.76 (0.75–0.76)	0.72 (0.71–0.73)	0.63 (0.62–0.64)	0.92 (0.91–0.93)	0.88 (0.87–0.89)
Valve disease	0.85 (0.85–0.86)	1.24 (1.23–1.25)	0.86 (0.85–0.86)	1.19 (1.18–1.20)	0.85 (0.84–	

T2DM, type 2 diabetes mellitus.

Using data from the 2021 Census, we were able to estimate the number of people with fibrotic conditions as of 2022. It is estimated that more than 7.2 million people in the UK have at least one fibrotic condition, with more than 3 million people estimated to have multiple organ fibrosis (table 4). Intestinal/pancreatic fibrosis and skeletal fibrosis are both estimated to affect close to 3 million people, respectively.

**Table 4:** Estimated prevalence and number of adults in England with fibrotic conditions as of 2022

	2022	
	**Estimated prevalence %**	**Estimated number of people**
Any organ fibrosis	19.95 (19.92–19.98)	7 265 021
Single organ fibrosis	15.39 (15.39–15.41)	5 604 445
Multiple organ fibrosis	8.51 (8.50–8.53)	3 099 014
Atherosclerosis	0.56 (0.56–0.57)	203 930
Biliary fibrosis	1.00 (0.99–1.00)	364 161
Blood vessel fibrosis	1.22 (1.22–1.23)	444 277
Cardiomyopathy	3.00 (2.99–3.01)	1 092 484
Insulin resistant T2DM	1.17 (1.17–1.18)	426 068
Integumentary fibrosis	3.84 (3.83–3.85)	1 398 380
Intestinal/pancreatic fibrosis	8.15 (8.14–8.17)	2 967 916
Liver fibrosis	1.63 (1.62–1.64)	593 583
Lymphatic fibrosis	1.63 (1.63–1.64)	109 248
Reproductive fibrosis	0.30 (0.30–0.30)	786 588
Lung fibrosis	2.16 (2.15–2.17)	593 583
Skeletal fibrosis	8.01 (7.99–8.03)	2 916 933
Systemic fibrosis	0.58 (0.57–0.58)	211 213
Treatment-resistant hypertension	0.24 (0.24–0.24)	87 398
Urinary fibrosis	0.76 (0.75–0.76)	276 762
Valve disease	1.24 (1.23–1.25)	451 560

Employing data from the 2021 Census.

T2DM, type 2 diabetes mellitus.

Hazards of mortality (adjusted for age and sex) increased with respect to increasing number of fibrotic conditions. The hazard for mortality in people with two fibrotic conditions was 2.90 (95% CI 2.87 to 2.93) when compared with people with single organ fibrosis. The mortality hazard in people with four fibrotic conditions was 8.79 (95% CI 8.61 to 8.99) (table 5).

**Table 5:** HR of mortality comparing risk in people with multiple fibrotic conditions with those with single organ fibrosis, adjusted for age and sex

	HR (95% CI)
1	Ref
2	2.90 (2.87 to 2.93)
3	5.22 (5.14 to 5.30)
≥4	8.79 (8.61 to 8.99)

## Discussion

The prevalence of fibrotic conditions and multiple organ fibrosis increased from 2012 to 2022. Fibrotic conditions were more common in females and prevalence increased with increasing age. The number of primary care consultations varied with respect to proximity to diagnosis; however, people with multiple fibrotic conditions accessed healthcare to a greater extent compared with people with single organ fibrosis and were at a greater risk of mortality.

To our knowledge, this is the first publication which has looked to describe the epidemiology of single and multiple organ fibrosis. A single study previously defined the prevalence of single and multiple organ fibrosis in England in 2015.^12^ Our findings with respect to the point prevalence in 2015 are similar to previously published figures. It is known that people are living longer and that fibrosis is associated with older age, which may partially explain the observation of rising prevalence from 2012 to 2022. However, prevalence of fibrosis increased from 2012 to 2022 across all age groups. Fibrosis is also associated with lifestyle factors including smoking, obesity and alcohol consumption; lifestyle factors and trends may also play an important role in the changing prevalence. Ultimately, it is not known what specific factors or combination of factors have contributed to the increase in prevalence; however, it is important that these be further understood, and prevention strategies as well as opportunities for screening and earlier identification be explored.

It is known that multiple long-term conditions increase burden on patients and health services.^21^ Therefore, it is important to understand the relationship between multiple fibrotic conditions, as such we looked to understand how many people had multiple fibrotic conditions and which conditions commonly co-occurred with one another. It is important to further understand whether a particular mechanism, systemic condition or environmental/genetic risk factors contribute towards the development of multiple fibrotic conditions in the same individual. This would provide evidence, informing practical strategies to assess the changing risk of a patient progressing from single organ fibrosis to multiple organ fibrosis.

Given the greater use of healthcare services by people with multiple organ fibrosis compared with people with single organ fibrosis, it is important to highlight potential implications of this. Given the greater burden on primary care resources as well as the impact this has on people with fibrotic multimorbidity, it is important that primary care consultations best serve these people. Earlier intervention by general practitioners leading to earlier diagnosis inevitably provides an opportunity for people to receive appropriate care at the earliest opportunity. It is also important that general practitioners are equipped with the resources to help these people with multiple fibrotic conditions, which often present as complex cases.

The prevalence of single and multiple organ fibrosis was greater in females compared with males. Four of the five most prevalent fibrotic conditions were more prevalent in females compared with males, likely contributing towards this observation. Sex differences have been previously observed with respect to fibrosis pathology, progression and treatment response.^22–24^ It is noteworthy that the prevalence of fibrotic conditions in women aged 40–44 years (12.48%) was lower than in females aged 65–69 years (25.58%), potentially due (in part) to change in risk with respect to menopause and loss of the potential protective effect of oestrogens (as well as other ovarian hormones).^22 25–27^

Fibrotic conditions which were more common in males compared with females included cardiomyopathy, lung and liver fibrosis. Cardiomyopathy is known to be more common in males; it has been hypothesised that this is due to the role of testosterone.^28^ It has been previously shown that high levels of testosterone promote inflammation in myocarditis which may lead to fibrosis.^29–31^ Emerging evidence suggests that mosaic loss of Y chromosome (mLOY) may have a role in the development and progression of fibrosis: mouse models having shown that cardiac macrophages with mLOY exhibit profibrotic pathologies, in turn, impacting cardiac function^32^; and a study using data from UK Biobank suggested that mLOY increases the risk of idiopathic pulmonary fibrosis,^33^ although these data need confirmation in disease-specific cohorts.

This work serves as a basis to aid hypothesis generation, to enable other researchers to use the estimates and other study findings in grant applications and to highlight the importance of fibrotic conditions. It is currently unknown whether the co-occurrence of fibrotic conditions is due to shared genetics, molecular pathways and/or risk factors. Therefore, it is important that future research is conducted and that the potential of a shared fibrotic mechanism across organs be explored. Additionally, we believe that the estimates of fibrotic conditions will be important for charities to support campaigns to increase awareness. Finally, by exploring the healthcare utilisation patterns of people with single and multiple organ fibrosis we highlight the increased use of primary care services by people with multiple fibrotic conditions as well as earlier opportunities for disease identification.

There are many strengths of this study, including the use of large scale, representative primary care data linked with secondary care records. Fibrotic conditions were identified in either primary or secondary care records, allowing for greater case identification, as it was previously observed that some fibrotic conditions were more commonly diagnosed in hospital, with others in general practice.^12^ Our prevalence estimates and the estimated number of people with fibrotic conditions are representative of the population of England as of 2022. We stratified these estimates by sex, age and region providing a thorough exploration; however, we were unable to stratify by deprivation measure or ethnicity. Regional estimates were standardised to the sex and age distribution of the general population of England, therefore improving external validity. However, there are also limitations. We could not adjust for ethnicity or deprivation as these measures are not available for the denominator population; these are important variables however which should be considered within the research context. Lifestyle factors such as smoking status, body mass index and alcohol consumption were not included in the analyses because complete and consistent data were not available across the study population. As these factors are associated with fibrosis risk, the reported HRs may be influenced by residual confounding. Consequently, the findings should be interpreted as measures of association rather than evidence of causality. The definitions of fibrotic conditions were derived from a previous publication; however, some of these conditions may only be fibrotic at a certain disease stage, it is for this reason that common conditions of hypertension and diabetes were more strictly defined in this publication. We relied on accurate clinical coding of conditions; there may have been people included in our denominator who had a fibrotic condition without coded evidence and therefore our estimates may be underestimated.

## Conclusion

The prevalence of single and multiple organ fibrosis has increased substantially over a decade, with no signs of plateau. Current estimates suggest that almost 11% of the UK population have at least one fibrotic condition. Healthcare utilisation increased in the years preceding diagnosis of fibrosis and plateaued post diagnosis, revealing missed opportunities for earlier diagnosis. Multiple organ fibrosis increased mortality risk compared with single organ fibrosis. As the prevalence of multiple organ fibrosis continues to increase, healthcare burden will also inevitably increase. Research regarding the potential of decreasing the risk of progressing from single to multiple organ fibrosis is severely lacking and should be prioritised.

## Supplementary material

10.1136/bmjopen-2025-113800 Supplementary file 1

## Data Availability

Data may be obtained from a third party and are not publicly available.
