# Supplementary material for: Temporal trends in single and multiple organ fibrosis prevalence and primary care consultations: a population-based cohort study of 5.8 million individuals in England
Source: BMJ Open. 2026 Sep 22;16(9):e113800. doi: 10.1136/bmjopen-2025-113800 (PMC13599825; doi:10.1136/bmjopen-2025-113800)
Supplement: Supplementary file 1 [file bmjopen-2025-113800.reviewer_comments.pdf]

PEER REVIEW HISTORY

BMJ Open publishes all reviews undertaken for accepted manuscripts. Reviewers are asked to complete a checklist review form and are provided with free text boxes to elaborate on their assessment. These free text comments are reproduced below.

ARTICLE DETAILS

Title (Provisional)

Temporal trends in single and multiple organ fibrosis prevalence and primary care consultations: A population based cohort study of 5·8 million individuals in England

Authors

Massen, Georgie May; Jenkins, Gisli; Allen, Richard J; Stewart, Iain; Scotton, Chris J; Chambers, Rachel; Longhurst, Hilary; Wain, Louise V; Navaratnam, Vidya; Quint, Jennifer K; Consortium, DEMISTIFI

VERSION 1 - REVIEW

**Reviewer**	**1**
**Name**	**Dack, Kyle**
**Affiliation**	**University of Bristol, MRC Integrative Epidemiology Unit**
**Date**	**20-Jan-2026**
**COI**	**None**

Statistical review summary:

This is primarily a descriptive analysis of trends in the UK. The statistical analysis looks reasonable, and the results are interpreted appropriately. I was a little unsure regarding the denominator for prevalence – only one overall denominator is reported, but shouldn't it vary by year and sex? Other than this I have only minor suggestions to improve the presentation.

Abstract:

1. Line 50 indicates that for this particular model, fibrotic conditions are the exposure, and the outcome is risk of death. I suggest a brief clarification that therefore death was the secondary (?) outcome.

Introduction:

2. Line 94: I am unfamiliar with the condition so cannot comment on the biological rationale or context, but generally it is clear and sets out a rationale for the study. My only suggestion/concern is that while the primary aim (patterns of fibrotic conditions) is described, there is not mention of what seems to be a secondary aim of estimating the association between fibrotic conditions and death.

Methods:

3. Line 112: A minor suggestion to improve clarity – add “while the” before “date of study exit” to ensure a clear separation of the two definitions in this sentence.

4. Line 114: While the outcome of interest is defined clearly from a theoretical standpoint, it would be beneficial to clarify the practical elements of determining the outcome. The github link contains codelists with two key columns – medcodeid and term – were both of these searched, or only the codeid? Were there specific fields in the datasets which were searched for this? I am unsure how clean the data is, but was any fuzzy matching required to account for typos etc – or other QC steps performed?

5. Outcomes: There seem to be two other related outcomes – the number of fibrotic conditions, and death. For completeness I recommend adding these. When counting the number of fibrotic conditions, was it a simple summing of matched diagnoses, or was there any deduplication necessary, e.g. to account for overlapping diagnoses which may reflect the same underlying condition.

6. Line 147: I suggest prior to the statistical analysis adding a section defining how HCRU was measured, e.g. what it includes and what is not included, so readers avoid misinterpreting the statistics. There are also other variables which would benefit from this, e.g. co-occuring fibrotic conditions, and the previously mentioned mortality and number of fibrotic conditions. Perhaps this could be contained in a separate “variables” section, if these are not considered outcomes?

Results

7. Tables 4 and 5: These are missing table labels. Table 5 would especially benefit from more details, e.g. the model N, the outcome being modelled, what 1/2/3/4 refer to (number of conditions), the adjusted covariates, generally making it a full model results table.

8. Line 170: It doesn’t look like there is inclusion/exclusion criteria related to length of followup so I was unsure what timepoint this denominator refers to. Does the denominator vary year on year? If so, it would be necessary to use different denominators in the calculation of each year’s annual prevalence. If the denominator does change, I suggest reporting this in-text (e.g. the min-max) and adding the denominators to supplementary table 1, or another suitable location.

9. Table 3: This table presents prevalence estimates stratified by type and sex. It would be helpful to clarify whether the denominator is also stratified accordingly, or whether a common population denominator is used (and whether this is year-specific). I recommend specifying the denominator in each table or figure that reports prevalence, because while this may look repetitive, it would aid interpretability and prevent misinterpretation by readers.

10. Line 183-185: For this comparison to be fair, it would require the denominator to be sex-specific. I imagine sex-specific denominators were used, but this is currently unreported in

the methods or results, leading to ambiguity. Again it could be something which is possible to add to a corresponding table or supplementary table.

11. Figure 1. It looks like there are three groupings of categories which should have an equal total prevalence:

(a) “at least 1 fibrotic condition”

(b) the sum of “single organ fibrosis”, “two fibrotic conditions”, “three fibrotic conditions”, “four fibrotic conditions”, and “five fibrotic conditions”

(c) the sum of “single organ fibrosis” and “multiple organ fibrosis”.

But looking at the figure, these do not seem equal. Perhaps I have misinterpreted the categories, but I suggest validating that these sum as would be expected.

**Reviewer**	**2**
**Name**	**Ratner, Leah**
**Affiliation**	**Brigham and Women's Hospital, Division of Global Health Equity**
**Date**	**23-Jun-2026**
**COI**	**None**

This study does not feel like it is clinically relevant. Consider re-doing this analysis with a less heterogeneous group of diseases. Linking "fibrotic" conditions together in this way -- is too heterogeneous and does not make sense as a finding. Consider re-doing with the help of a clinician.

**Reviewer**	**3**
**Name**	**Dai, Huaping**
**Affiliation**	**Beijing Institute of Respiratory Medicine, Department of Respiratory and Critical Care Medicine, Beijing Chao-Yang Hospital**
**Date**	**21-Jul-2026**
**COI**	**None**

This ten-year epidemiological analysis was based on nationally representative primary and secondary electronic health record databases in the UK, covering nearly 5.8 million individuals with fibrosis and a baseline population of over 18 million participants. The study addresses a timely public health hotspot concerning multimorbidity and the population burden of fibrotic diseases. With a remarkably large sample size and comprehensive

stratified analyses, the work possesses substantial clinical healthcare policy implications. Nevertheless, several methodological and interpretative limitations remain to be addressed.

1.Only the median number of primary care consultations before diagnosis was directly reported for patients with single-organ versus multi-organ fibrosis (14 vs 21 visits). No intergroup nonparametric tests such as the rank-sum test were performed, and no P values or confidence intervals were provided. As a result, the statistical significance of the difference in consultation burden between the two groups could not be verified.

2.The Cox regression model omitted critical confounding variables, which limits the validity of risk-related conclusions.The model was only adjusted for age and sex, while key confounders strongly linked to fibrosis were not incorporated, including smoking status, obesity, alcohol consumption, socioeconomic deprivation, ethnicity, and coexisting chronic conditions (excluding fibrotic disorders). Although the manuscript acknowledged deprivation and ethnicity as important potential confounders that could not be adjusted due to database limitations, it failed to quantify the direction of bias introduced by this omission in the statistical methods or limitations sections.

3.Standardization approaches for stratified prevalence estimates by region, age and sex were not described. The manuscript did not state whether standardization to the UK population structure was conducted. Crude prevalence rates fail to eliminate confounding effects from interregional disparities in population age composition, reducing the reliability of regional comparative conclusions.

4.The grouping strategy for patients with multiple fibrotic organs was overly aggregated. Participants with five or more fibrotic organs were combined into a single category, without separate analyses of prevalence and mortality risk gradients for patients with 5, 6 or more affected organs. This aggregation obscures the extreme disease burden observed in patients with severe multi-organ fibrosis. It would be better to clearly justify this combined grouping strategy and report the proportional distribution of participants across each count of fibrotic organs.

VERSION 1 - AUTHOR RESPONSE

Reviewer: 1

Dr. Kyle Dack, University of Bristol

Comments to the Author:

Statistical review summary:

This is primarily a descriptive analysis of trends in the UK. The statistical analysis looks reasonable, and the results are interpreted appropriately. I was a little unsure regarding the denominator for prevalence – only one overall denominator is reported, but shouldn’t it vary by year and sex? Other than this I have only minor suggestions to improve the presentation.

Thank you for highlighting this. We have now added to table 1 and additionally made table 5) in the supplementary materials, which details the size of the denominator by sex and year. The denominator we refer to is the total number of people included in the denominator file, we hope that adding signposting to the updated supplementary figure aids interpretation. We also clarify in the methods "the denominator was defined per year and further stratified by both age and sex where necessary".

Abstract:

1. Line 50 indicates that for this particular model, fibrotic conditions are the exposure, and the outcome is risk of death. I suggest a brief clarification that therefore death was the secondary (?) outcome.

Thank you for this helpful suggestion. We agree that the original wording did not clearly distinguish the prevalence analysis from the mortality analysis. We have revised the abstract to explicitly state that the study first estimated the prevalence of single- and multiple-organ fibrosis and then performed a secondary analysis examining the association between the time-updated number of fibrotic conditions and subsequent risk of death using Cox proportional hazards models adjusted for age and sex. The abstract now reads "The primary analysis estimated the prevalence of single- and multiple-organ fibrosis. A secondary analysis used Cox proportional hazards models to investigate the association between the time-updated number of fibrotic conditions and risk of death, adjusting for age and sex. "

Introduction:

2. Line 94: I am unfamiliar with the condition so cannot comment on the biological rationale or context, but generally it is clear and sets out a rationale for the study. My only suggestion/concern is that while the primary aim (patterns of fibrotic conditions) is described, there is not mention of what seems to be a secondary aim of estimating the association between fibrotic conditions and death.

Thank you, we have rewritten the final paragraph of the introduction, to now read "Given the previously observed high prevalence of fibrotic conditions and the association between the number of fibrotic conditions and mortality, it is imperative to better understand the burden of fibrotic disease. This will provide evidence to support further research investment as well as healthcare service provision."

Methods:

3. Line 112: A minor suggestion to improve clarity – add "while the" before "date of study exit" to ensure a clear separation of the two definitions in this sentence.

Thank you, we have now added this, improving clarity.

4. Line 114: While the outcome of interest is defined clearly from a theoretical standpoint, it

would be beneficial to clarify the practical elements of determining the outcome. The github link contains codelists with two key columns – medcodeid and term – were both of these searched, or only the codeid? Were there specific fields in the datasets which were searched for this? I am unsure how clean the data is, but was any fuzzy matching required to account for typos etc – or other QC steps performed?

Thank you, we have added the following clarification: "Each codelist comprised standardised clinical codes (medcodeid/ ICD10) and their corresponding clinical descriptors. Outcomes were identified through exact matching of the coded medcodeid/ICD10 value recorded in the CPRD Aurum observation dataset or the HES APC dataset."

5. Outcomes: There seem to be two other related outcomes – the number of fibrotic conditions, and death. For completeness I recommend adding these. When counting the number of fibrotic conditions, was it a simple summing of matched diagnoses, or was there any deduplication necessary, e.g. to account for overlapping diagnoses which may reflect the same underlying condition.

Thank you, we have added the following clarification. "Secondary outcomes of interest were the number of fibrotic conditions (specifically the number of effected organ/disease groups, thereby removing the potential for double counting of overlapping conditions or conditions effecting the same organ) and mortality (defined using provided date of death by ONS)."

6. Line 147: I suggest prior to the statistical analysis adding a section defining how HCRU was measured, e.g. what it includes and what is not included, so readers avoid misinterpreting the statistics. There are also other variables which would benefit from this, e.g. co-occuring fibrotic conditions, and the previously mentioned mortality and number of fibrotic conditions. Perhaps this could be contained in a separate "variables" section, if these are not considered outcomes?

Thank you, we have added the following " Other included variables of interest were the co-occurrence of fibrotic conditions, specifically which effected organ/disease groups were observed in the same individual (if FMM was present). Healthcare utilisation trends were also explored, with the number of primary care consultations in the years prior to and post diagnosis of fibrotic conditions. Routine visits such as vaccinations and health checks did not contribute towards these counts." Under the proposed heading.

Results

7. Tables 4 and 5: These are missing table labels. Table 5 would especially benefit from more details, e.g. the model N, the outcome being modelled, what 1/2/3/4 refer to (number of conditions), the adjusted covariates, generally making it a full model results table.

Thank you, the table legends were only included at the end of the main manuscript, we have

now updated the legends and ensured they are present in all associated files.

8. Line 170: It doesn't look like there is inclusion/exclusion criteria related to length of followup so I was unsure what timepoint this denominator refers to. Does the denominator vary year on year? If so, it would be necessary to use different denominators in the calculation of each year's annual prevalence. If the denominator does change, I suggest reporting this in-text (e.g. the min-max) and adding the denominators to supplementary table 1, or another suitable location.

Thank you, you are correct the denominator changed each year, we have now detailed the values in supplementary table one as you suggest and clarified in the methods "To be eligible for inclusion in the denominator, people had to be either male or female (to ensure data completeness), over the age of 18 and enrolled at a practice which was eligible for linkage to HES APC, the denominator was generated per year and further stratified by age and sex where necessary."

9. Table 3: This table presents prevalence estimates stratified by type and sex. It would be helpful to clarify whether the denominator is also stratified accordingly, or whether a common population denominator is used (and whether this is year-specific). I recommend specifying the denominator in each table or figure that reports prevalence, because while this may look repetitive, it would aid interpretability and prevent misinterpretation by readers.

Thank you, all analyses used year specific and stratified denominators (by either age or sex or both) where necessary. We now include supplementary table 5 which details the size of the denominators for this analysis.

10. Line 183-185: For this comparison to be fair, it would require the denominator to be sex-specific. I imagine sex-specific denominators were used, but this is currently unreported in the methods or results, leading to ambiguity. Again it could be something which is possible to add to a corresponding table or supplementary table.

Thank you, we have now clarified this in the text, alongside additions to supplementary tables one and five respectively.

11. Figure 1. It looks like there are three groupings of categories which should have an equal total prevalence:

(a) "at least 1 fibrotic condition"

(b) the sum of "single organ fibrosis", "two fibrotic conditions", "three fibrotic conditions", "four fibrotic conditions", and "five fibrotic conditions"

(c) the sum of "single organ fibrosis" and "multiple organ fibrosis".

But looking at the figure, these do not seem equal. Perhaps I have misinterpreted the categories, but I suggest validating that these sum as would be expected.

Thank you for highlighting this point. We agree that, without further clarification, there may be an expectation that these prevalence estimates should sum to the same overall prevalence.

However, the estimates shown in Figure 1 were calculated using different denominators as a result of the censoring applied to each analysis. In particular, individuals who developed multiple-organ fibrosis were no longer considered at risk of single-organ fibrosis and were therefore removed from the denominator for the single-organ fibrosis analysis. Consequently, the prevalence estimates for single-organ fibrosis, multiple-organ fibrosis and the number of fibrotic conditions are not mutually exclusive components calculated from a common denominator and therefore are not expected to sum to the overall prevalence of having at least one fibrotic condition. We have revised the figure legend and Methods to make this clearer. The methods now states: "As a result of this censoring, the denominators used to estimate the prevalence of fibrosis, single organ fibrosis and multiple organ fibrosis differed; consequently, these prevalence estimates are not expected to sum to the overall prevalence of fibrosis affecting at least one organ."

Reviewer: 2

Dr. Leah Ratner, Brigham and Women's Hospital, Harvard Medical School

Comments to the Author:

This study does not feel like it is clinically relevant. Consider re-doing this analysis with a less heterogeneous group of diseases. Linking "fibrotic" conditions together in this way -- is too heterogeneous and does not make sense as a finding. Consider re-doing with the help of a clinician.

Thank you for raising this important point. We respectfully disagree that the grouping of fibrotic conditions in this analysis lacks clinical rationale. The conditions included in this study were not selected solely by the study authors or grouped together on the basis of electronic health record coding. Rather, they were identified through our preceding Delphi consensus study (Massen, G.M., Allen, R.J., Leavy, O.C., Selby, N.M., Aithal, G.P., Oliver, N., Parfrey, H., Wain, L.V., Jenkins, G., Stewart, I. and Quint, J.K., 2023. Classifying the unclassifiable—a Delphi study to reach consensus on the fibrotic nature of diseases. *QJM: An International Journal of Medicine*, *116*(6), pp.429-435.), in which clinicians with relevant expertise were asked to establish which conditions should be considered fibrotic. This work was undertaken specifically to provide a clinically informed foundation for subsequent epidemiological investigation of fibrosis across organ systems.

The present study forms part of a broader programme of research investigating fibrotic multimorbidity and was designed to address an important initial epidemiological question: how frequently do conditions characterised by fibrosis occur individually and across multiple organ systems at a population level? We recognise that the conditions included are clinically

heterogeneous, and we agree that this is an important consideration when interpreting the findings. However, the purpose of the study was not to suggest that these diseases constitute a single homogeneous clinical entity. Rather, by examining fibrosis across organ systems, we aimed to quantify the population burden and co-occurrence of conditions characterised by fibrotic processes and to identify patterns that may warrant more focused clinical and mechanistic investigation.

Clinical expertise has been integral to the development of this work. Multiple clinicians were involved in the preceding consensus work and in the design and interpretation of the current study. Furthermore, this analysis forms part of a wider programme of research into fibrotic multimorbidity [https://gtr.ukri.org/projects?ref=MR%2FW014491%2F1], within which subsequent work is examining more clinically and mechanistically specific groupings of fibrotic disease.

Reviewer: 3

Dr. Huaping Dai, Beijing Institute of Respiratory Medicine

Comments to the Author:

This ten-year epidemiological analysis was based on nationally representative primary and secondary electronic health record databases in the UK, covering nearly 5.8 million individuals with fibrosis and a baseline population of over 18 million participants. The study addresses a timely public health hotspot concerning multimorbidity and the population burden of fibrotic diseases. With a remarkably large sample size and comprehensive stratified analyses, the work possesses substantial clinical healthcare policy implications. Nevertheless, several methodological and interpretative limitations remain to be addressed.

Thank you for your helpful comments which have strengthened our proposed work.

1.Only the median number of primary care consultations before diagnosis was directly reported for patients with single-organ versus multi-organ fibrosis (14 vs 21 visits). No intergroup nonparametric tests such as the rank-sum test were performed, and no P values or confidence intervals were provided. As a result, the statistical significance of the difference in consultation burden between the two groups could not be verified.

Thank you for this comment. The healthcare resource utilisation analysis was intended to provide a descriptive characterisation of patterns before and after diagnosis, rather than to formally test differences in utilisation between the single- and multiple-organ fibrosis groups. We therefore did not undertake hypothesis testing at individual time points, as this was outside the primary aims of the study and would introduce multiple comparisons across the

longitudinal observation period. However, we agree that providing information on the variability of consultation burden improves the interpretation of these findings. We have therefore added interquartile ranges (IQRs) to the reported medians and revised Figure 3A to display the IQRs for primary care consultations. We have also revised the text to make clear that these comparisons are descriptive and do not imply statistical significance.

We have now revised the text to read: Healthcare resource utilisation increased in the years leading up to diagnosis of either single organ or multiple organ fibrosis (**Figure 3A**). In the year preceding diagnosis of single organ fibrosis, the median number of primary care consultations was 14 (IQR: 8-23), whilst the median number over the same period in people with multiple organ fibrosis was 21 (IQR: 12-32) consultations. Healthcare utilisation subsequently declined following diagnosis in both groups.

2.The Cox regression model omitted critical confounding variables, which limits the validity of risk-related conclusions. The model was only adjusted for age and sex, while key confounders strongly linked to fibrosis were not incorporated, including smoking status, obesity, alcohol consumption, socioeconomic deprivation, ethnicity, and coexisting chronic conditions (excluding fibrotic disorders). Although the manuscript acknowledged deprivation and ethnicity as important potential confounders that could not be adjusted due to database limitations, it failed to quantify the direction of bias introduced by this omission in the statistical methods or limitations sections.

Thank you for this important comment. We agree that additional adjustment for lifestyle and demographic factors would strengthen causal inference. The present study was designed as a population-based epidemiological analysis to describe associations rather than estimate causal effects. Age and sex were included as a priori confounders because they are strongly associated with both fibrosis risk and multimorbidity. Although smoking status, obesity, alcohol consumption, socioeconomic deprivation, ethnicity and non-fibrotic comorbidities are also potential confounders, these variables were unavailable or incompletely recorded for the study population and therefore could not be incorporated consistently into the regression models without substantially reducing the analysable cohort and introducing selection bias. We have expanded the Limitations section to explicitly discuss the potential for residual confounding and to clarify that the observed hazard ratios may be over- or underestimated depending on the distribution of these unmeasured factors across exposure groups. Accordingly, the findings should be interpreted as associations rather than causal estimates.

We have strengthened our limitations section to now read "Lifestyle factors such as smoking status, BMI and alcohol consumption were not included in the analyses because complete and consistent data were not available across the study population. As these factors are associated with fibrosis risk, the reported HRs may be influenced by residual confounding. Consequently, the findings should be interpreted as measures of association rather than evidence of causality."

3.Standardization approaches for stratified prevalence estimates by region, age and sex were not described. The manuscript did not state whether standardization to the UK population structure was conducted. Crude prevalence rates fail to eliminate confounding effects from interregional disparities in population age composition, reducing the reliability of regional comparative conclusions.

Thank you for highlighting this important point. The regional prevalence estimates in the original manuscript were crude and therefore did not account for differences in population structure between regions. We have now repeated the regional analyses using direct age- and sex-standardisation to the 2021 England Census population. Age- and sex-specific prevalence estimates were calculated within each region and weighted according to the corresponding distribution of the standard population. We have replaced the crude regional estimates in Table 1 with age- and sex-standardised prevalence estimates and corresponding 95% confidence intervals and have updated the Methods and Results accordingly. Regional variation remained following standardisation, with the North East having the highest standardised prevalence of at least one fibrotic condition, single organ fibrosis and multiple organ fibrosis in 2022.

Methods: "To account for differences in population structure between regions, regional prevalence estimates were directly standardised by age and sex to the 2021 England Census population. Age and sex-specific prevalence estimates were calculated within each region and weighted according to the corresponding age and sex-specific distribution of the standard population."

Results: "Age and sex-standardised prevalence varied across regions of England (**Table 1**). In 2022, the prevalence of at least one fibrotic condition was highest in the North East (26.54%, 95% CI : 26.40-26.69) and lowest in London (29.13%, 95% CI: 20.0A7-20.18). A similar pattern was observed for single organ fibrosis, the prevalence ranged from 18.04% (95%CI: 17.99-18.10) in London to 23.10% (95% CI: 22.95-23.25) in the North East, Multiple organ fibrosis showed greater regional variation, the highest prevalence was observed in the North East (10.80%, 95% CI: 10.69-10.90_, compared with 7.51% (95% CI: 7.38-7.64 in the East Midlands, where prevalence was lowest. Age and sex-standardised prevalence increased between 2012 and 2022 across all regions for each of the three fibrosis outcomes."

4.The grouping strategy for patients with multiple fibrotic organs was overly aggregated. Participants with five or more fibrotic organs were combined into a single category, without separate analyses of prevalence and mortality risk gradients for patients with 5, 6 or more affected organs. This aggregation obscures the extreme disease burden observed in patients with severe multi-organ fibrosis. It would be better to clearly justify this combined grouping strategy and report the proportional distribution of participants across each count of fibrotic organs.

Thank you for raising this, due to the small number of individuals with this number of conditions it was deemed appropriate to group these conditions. We have clarified this in the methods.

"(1 organ, 2, 3, >=4, grouped as four or more due to low numbers)"

VERSION 2 - REVIEW

**Reviewer**	**1**
**Name**	**Dack, Kyle**
**Affiliation**	**University of Bristol, MRC Integrative Epidemiology Unit**
**Date**	**26-Aug-2026**
**COI**	

No further comments or queries - the manuscript has clear methods, statistics, and reporting is strong.
