# Supplementary material for: Temporal trends in single and multiple organ fibrosis prevalence and primary care consultations: a population-based cohort study of 5.8 million individuals in England
Source: BMJ Open. 2026 Sep 22;16(9):e113800. doi: 10.1136/bmjopen-2025-113800 (PMC13599825; doi:10.1136/bmjopen-2025-113800)
Supplement: Supplementary file 2 [file bmjopen-16-9-s001.docx]

**Table 1 | Prevalence of fibrosis, single organ fibrosis and multiple organ fibrosis between 2012 and 2022**

|  | **Prevalence, % (95% CI)** | | | **Denominator (n)** | | |
| --- | --- | --- | --- | --- | --- | --- |
|  | **At least one fibrotic condition** | **Single organ fibrosis** | **Multiple organ fibrosis** | **At least one fibrotic condition** | **Single organ fibrosis** | **Multiple organ fibrosis** |
| **2012** | 13.23  (13.21–13.25) | 12.94  12.92-12.97) | 4.78  (4.76–4.79) | 8,110,043 | 8,083,128 | 8,925,336 |
| **2013** | 14.33  (14.31-14.36) | 13.44  (13.42-13.47) | 5.22  (5.21-5.23) | 8,189,791 | 8,105,688 | 8,994,496 |
| **2014** | 15.37  (15.35-15.40) | 13.92  (13.90-13.95) | 5.67  (5.66-5.69) | 8,264,665 | 8,125,697 | 9,132,717 |
| **2015** | 16.32  (16.30-16.35) | 14.37  (14.34-14.39) | 6.11  (6.10-6.13) | 8,419,558 | 8,227,327 | 9,313,788 |
| **2016** | 17.18  (17.15-17.20) | 14.75  (14.72-14.77) | 6.56  (6.54-6.57) | 8,619,919 | 8,374,194 | 9,503,501 |
| **2017** | 17.94  (17.91-17.97) | 15.05  (15.03-15.08) | 7.00  (6.99-7.02) | 8,803,834 | 8,504,700 | 9,704,560 |
| **2018** | 18.63  (18.60 – 18.66) | 15.32  (15.29-15.34) | 7.45  (7.43-7.47) | 8,976,328 | 8,625,012 | 9,880,749 |
| **2019** | 19.38  (19.35-19.40) | 15.62  (15.60-15.65) | 7.94  (7.92-7.96) | 9,018,158 | 8,617,020 | 9,949,521 |
| **2020** | 19.70  (19.68-19.73) | 15.67  (15.65-15.69) | 8.21  (8.20-8.23) | 9,071,304 | 8,637,408 | 9,959,823 |
| **2021** | 19.99  (19.96-20.01) | 15.39  (15.36-15.41) | 8.35  (8.33-8.37) | 9,071,310 | 8,608,608 | 10,012,472 |
| **2022** | 19.95  (19.92–19.98) | 15.39  (15.39-15.41 | 8.51  (8.50-8.53) | 8,925,606 | 8,444,134 | 9,677,405 |

**Table 2 | Prevalence of fibrotic conditions by count between 2012 and 2022.**

|  | **Prevalence, % (95% CI)** | | | |
| --- | --- | --- | --- | --- |
|  | **Two fibrotic conditions** | **Three fibrotic conditions** | **Four fibrotic conditions** | **Five plus fibrotic conditions** |
| **2012** | 3.12  (3.11-3.13) | 0.82  (0.81-0.82) | 0.21  (0.21-0.22) | 0.07  (0.07-0.07) |
| **2013** | 3.37  (3.36-3.38) | 0.92  (0.91-0.92) | 0.25  (0.24-0.25) | 0.09  (0.08-0.09) |
| **2014** | 3.62  (3.61-3.63) | 1.02  (1.01-1.03) | 0.29  (0.28-0.29) | 0.10  (0.10-0.10) |
| **2015** | 3.86  (3.85-3.88) | 1.12  (1.11-1.13) | 0.32  (0.32-0.33) | 0.12  (0.12-0.12) |
| **2016** | 4.10  (4.08-4.11) | 1.23  (1.22-1.24) | 0.36  (0.36-0.37) | 0.14  (0.14-0.14) |
| **2017** | 4.33  (4.31-4.34) | 1.34  (1.33-1.35) | 0.41  (0.40-0.41) | 0.16  (0.16-0.16) |
| **2018** | 4.54  (4.52-4.55) | 1.45  (1.45-1.46) | 0.45  (0.45-0.46) | 0.19  (0.18-0.19) |
| **2019** | 4.76  (4.75-4.77) | 1.58  (1.57-1.59) | 0.51  (0.51-0.51) | 0.22  (0.21-0.22) |
| **2020** | 4.87  (4.85-4.88) | 1.65  (1.64-1.66) | 0.54  (0.54-0.55) | 0.24  (0.23-0.24) |
| **2021** | 4.89  (4.87-4.90) | 1.69  (1.68-1.70) | 0.57  (0.56-0.57) | 0.26  (0.25-0.26) |
| **2022** | 4.92  (4.90-4.93) | 1.74  (1.73-1.75) | 0.60  (0.59-0.60) | 0.28  (0.27-0.28) |

**Table 3 | Prevalence of fibrotic conditions, single organ fibrosis and multiple organ fibrosis, stratified by area.**

|  | **At least one fibrotic condition, % (95% CI)** | | | **Single organ fibrosis, % (95% CI)** | | | **Multiple organ fibrosis, % (95% CI)** | | |
| --- | --- | --- | --- | --- | --- | --- | --- | --- | --- |
|  | **2012** | **2017** | **2022** | **2012** | **2017** | **2022** | **2012** | **2017** | **2022** |
| **North East** | 20.78  (20.63-20.92) | 25.34  (25.18-25.49) | 27.66  (27.50-27.82) | 15.29  (15.15-15.42) | 17.95  (17.81-18.10) | 18.91  (18.75-19.06) | 6.55  (6.46-6.63) | 9.13  (9.03-9.24) | 11.51  (11.39-11.62) |
| **North West** | 18.51  (18.45-18.57) | 22.99  (22.93-23.05) | 24.43  (24.37-24.49) | 13.82  (13.76-13.87) | 16.18  (16.12-16.23) | 16.58  (16.53-16.64) | 5.66  (5.63-5.69) | 8.39  (8.35-8.43) | 10.16  (10.12-10.21) |
| **Yorkshire** | 17.58  (17.45-17.70) | 21.59  (21.45-21.72) | 21.91  (21.75-22.07) | 13.77  (13.65-13.90) | 16.05  (15.92-16.18) | 15.97  (15.82-16.12) | 4.90  (4.83-4.97) | 7.15  (7.06-7.23) | 8.35  (8.24-8.45) |
| **East Midlands** | 14.92  (14.77-15.07) | 18.75  (18.60-18.91) | 16.42  (16.24-16.59) | 11.95  (11.81-12.10) | 14.20  (14.05-14.35) | 12.68  (12.51-12.86) | 4.17  (4.09-4.26) | 6.17  (6.08-6.27) | 6.09  (5.98-6.21) |
| **West Midlands** | 16.89  (16.83-16.95) | 21.30  (21.23-21.36) | 23.35  (23.29-23.42) | 13.34  (13.29-13.40) | 15.71  (15.65-15.77) | 16.56  (16.50-16.63) | 4.68  (4.64-4.71) | 7.23  (7.19-7.28) | 9.08  (9.04-9.13) |
| **East of England** | 17.38  (17.26-17.50) | 21.95  (21.83-22.07) | 23.36  (23.23-23.49) | 13.57  (13.46-13.68) | 16.15  (16.03-16.27) | 16.66  (16.54-16.78) | 4.74  (4.67-4.80) | 7.27  (7.19-7.34) | 8.91  (8.82-9.00) |
| **London** | 13.11  (13.06-13.16) | 15.86  (15.81-15.91) | 16.24  (16.19-16.29) | 10.79  (10.74-10.84) | 12.18  (12.12-12.23) | 12.07  (12.03-12.12) | 3.75  (3.72-3.78) | 5.31  (5.28-5.34) | 6.12  (6.09-6.15) |
| **South East** | 16.12  (16.07-16.17) | 20.09  (20.04-20.15) | 21.84  (21.78-21.89) | 12.65  (12.60-12.70) | 14.91  (14.86-14.97) | 15.55  (15.50-15.61) | 4.46  (4.43-4.49) | 6.62  (6.59-6.65) | 8.37  (8.33-8.40) |
| **South West** | 17.70  (17.63-17.77) | 21.55  (21.48-21.63) | 23.27  (23.19-23.35) | 13.56  (13.49-13.63) | 15.77  (15.70-15.84) | 16.41  (16.33-16.48) | 5.18  (5.14-5.22) | 7.37  (7.33-7.42) | 9.16  (9.10-9.21) |

|  | **Prevalence of single organ fibrosis, % (95%CI)** | | | | **Prevalence of multiple organ fibrosis, % (95%CI)** | | | |
| --- | --- | --- | --- | --- | --- | --- | --- | --- |
|  | **Females** | | **Males** | | **Females** | | **Males** | |
|  | **2012** | **2022** | **2012** | **2022** | **2012** | **2022** | **2012** | **2022** |
| **18-24** | 1.02  (1.00-1.04) | 1.45  (1.43-1.47) | 1.42  (1.40-1.45) | 1.80  (1.78-1.83) | 0.11  (0.11-0.12) | 0.22  (0.21-0.23) | 0.09  (0.09-0.10) | 0.22  (0.21-0.23) |
| **25-29** | 4.67  (4.60-4.75) | 6.30  (6.22-6.39) | 3.92  (3.85-3.99) | 5.63  (5.55-5.70) | 0.63  (0.61-0.66) | 1.29  (1.25-1.33) | 0.31  (0.29-0.33) | 0.75  (0.72-0.78) |
| **30-34** | 6.44  (6.36-6.52) | 8.38  (8.30-8.47) | 3.87  (3.81-3.94) | 6.14  (6.06-6.21) | 0.95  (0.92-0.98) | 2.06  (2.01-2.10) | 0.43  (0.41-0.46) | 0.98  (0.95-1.01) |
| **35-39** | 8.63  (8.54-8.73) | 10.35  (10.25-10.44) | 4.75  (4.68-4.82) | 6.76  (6.68-6.83) | 1.40  (1.36-1.44) | 2.81  (2.76-2.86) | 0.67  (0.64-0.69) | 1.37  (1.33-1.40) |
| **40-44** | 10.44  (10.34-10.53) | 12.48  (12.37-12.58) | 6.35  (6.27-6.43) | 7.84  (7.76-7.93) | 1.94  (1.90-1.99) | 3.84  (3.78-3.90) | 1.10  (1.07-1.14) | 2.03  (1.99-2.08) |
| **45-49** | 11.95  (11.84-12.05) | 14.88  (14.76-15.00) | 8.33  (8.24-8.41) | 9.98  (9.88-10.08) | 2.54  (2.49-2.59) | 5.31  (5.23-5.38) | 1.71  (1.67-1.75) | 3.07  (3.01-3.12) |
| **50-54** | 13.82  (13.71-13.94) | 17.84  (17.72-17.97) | 11.28  (11.18-11.38) | 13.24  (13.13-13.34) | 3.51  (3.45-3.57) | 7.46  (7.38-7.55) | 2.72  (2.67-2.78) | 4.97  (4.91-5.04) |
| **55-59** | 16.23  (16.10-16.36) | 20.92  (20.79-21.05) | 14.55  (14.43-14.68) | 17.82  (17.70-17.95) | 4.94  (4.87-5.02) | 10.11  (10.02-10.21) | 4.41  (4.34-4.48) | 7.79  (7.71-7.88) |
| **60-64** | 18.87  (18.73-19.02) | 23.51  (23.36-23.66) | 18.49  (18.35-18.63) | 22.48  (22.34-22.63) | 6.54  (6.45-6.63) | 13.15  (13.03-13.26) | 6.70  (6.61-6.79) | 11.65  (11.54-11.76) |
| **65-69** | 21.49  (21.34-21.64) | 25.58  (25.41-25.75) | 21.93  (21.77-22.08) | 25.20  (25.03-25.37) | 8.63  (8.53-8.73) | 16.38  (16.24-16.52) | 9.48  (9.37-9.59) | 16.25  (16.11-16.39) |
| **70-74** | 24.21  (24.03-24.39) | 28.53  (28.35-28.72) | 25.04  (24.85-25.23) | 28.89  (28.70-29.09) | 11.92  (11.78-12.05) | 20.43  (20.27-20.58) | 13.67  (13.52-13.82) | 21.73  (21.56-21.89) |
| **75-79** | 26.21  (26.02-26.41) | 31.34  (31.13-31.54) | 27.20  (26.98-27.42) | 31.52  (31.31-31.74) | 15.26  (15.10-15.42) | 25.54  (25.36-25.71) | 17.53  (17.34-17.71) | 27.34  (27.15-27.53) |
| **80-84** | 27.77  (27.55-27.99) | 33.35  (33.09-33.61) | 28.07  (27.81-28.33) | 33.40  (33.11-33.70) | 18.16  (17.97-18.36) | 32.11  (31.88-32.34) | 20.79  (20.56-21.03) | 34.37  (34.11-34.63) |
| **85-89** | 28.39  (28.12-28.66) | 34.52  (34.20-34.84) | 28.43  (28.08-28.78) | 34.75  (34.36-35.14) | 20.12  (19.88-20.36) | 37.40  (37.11-37.69) | 22.54  (22.22-22.87) | 39.51  (39.16-39.86) |
| **90+** | 28.52  (28.20-28.85) | 34.03  (33.64-34.42) | 26.94  (26.44-27.44) | 34.24  (33.69-34.79) | 19.52  (19.24-19.80) | 40.64  (40.28-41.00) | 20.95  (20.50-21.41) | 41.82  (41.32-42.33) |
|  |  |  |  |  |  |  |  |  |
| **18+** | 14.11  (14.08-14.14) | 16.82  (16.78-16.85) | 11.75  (11.72-11.78) | 13.97  (13.94-14.01) | 5.10  (5.08-5.12) | 9.37  (9.34-9.39) | 4.44  (4.43-4.46) | 7.66  (7.64-7.68) |

**Table 4 | Prevalence of single and multiple organ fibrosis, stratified by sex and age.**

**Table 5 | Denominators employed by sex and age for single and multiple organ fibrosis.**

|  | **Denominator (n)** | | | | | | | |
| --- | --- | --- | --- | --- | --- | --- | --- | --- |
|  | **Single organ fibrosis** | | | | **Multiple organ fibrosis** | | | |
|  | **Females** | | **Males** | | **Females** | | **Males** | |
|  | **2012** | **2022** | **2012** | **2022** | **2012** | **2022** | **2012** | **2022** |
| **18-24** | 987,226 | 1,101,428 | 1,037,576 | 1,177,094 | 987,666 | 1,108,287 | 1,037,772 | 1,180,639 |
| **25-29** | 314,519 | 343,238 | 321,335 | 368,979 | 314,657 | 345,333 | 321,388 | 370,096 |
| **30-34** | 346,624 | 392,531 | 341,943 | 409,352 | 346,838 | 396,603 | 342,044 | 411,230 |
| **35-39** | 337,308 | 394,365 | 341,316 | 409,918 | 337,571 | 400,275 | 341,466 | 412,629 |
| **40-44** | 384,085 | 376,754 | 393,156 | 397,359 | 384,530 | 384,801 | 393,442 | 401,506 |
| **45-49** | 394,160 | 338,997 | 406,726 | 363,339 | 394,828 | 349,724 | 407,216 | 369,496 |
| **50-54** | 352,417 | 365,626 | 366,872 | 388,546 | 353,175 | 382,627 | 367,598 | 400,051 |
| **55-59** | 303,626 | 358,744 | 310,735 | 376,653 | 304,565 | 382,618 | 311,684 | 395,637 |
| **60-64** | 293,600 | 306,819 | 289,663 | 317,374 | 294,862 | 333,756 | 291,024 | 342,279 |
| **65-69** | 289,165 | 251,838 | 277,024 | 246,574 | 290,786 | 278,971 | 278,805 | 274,030 |
| **70-74** | 219,999 | 226,844 | 200,428 | 109,062 | 221,709 | 258,356 | 202,202 | 241,155 |
| **75-79** | 189,726 | 202,512 | 161,362 | 175,499 | 191,685 | 239,157 | 163,235 | 210,539 |
| **80-84** | 155,808 | 129,121 | 115,855 | 101,601 | 157,600 | 159,384 | 117,365 | 127,414 |
| **85-89** | 105,850 | 84,924 | 62,768 | 57,810 | 107,330 | 107,854 | 63,745 | 75,301 |
| **90+** | 76,246 | 57,017 | 30,431 | 28,216 | 77,278 | 72,275 | 30,903 | 36,845 |
|  |  |  |  |  |  |  |  |  |
| **18+** | 4,750,359 | 4,930,758 | 4,657,190 | 4,927,376 | 4,765,080 | 5,200,021 | 4,669,889 | 5,248,847 |
